# Development of a clinical prediction score for perioperative complications following metastatic spinal surgery (PERCOM) score

**DOI:** 10.1016/j.heliyon.2024.e25180

**Published:** 2024-01-26

**Authors:** Ryouhei Takeuchi, Kiyoshi Tarukado, Yoshihiro Matsumoto, Kei-ichiro Iida, Kazu Kobayakawa, Hirokazu Saiwai, Kenichi Kawaguchi, Yasuharu Nakashima

**Affiliations:** a Department of Orthopedic Surgery, Kyushu University Hospital, Fukuoka, Japan; b Department of Orthopedic Surgery, Fukushima Medical University School of Medicine, Fukushima, Japan

**Keywords:** Spinal metastasis, Spinal surgery, Perioperative complication, Prediction score

## Abstract

**Background:**

Spinal metastases can impair mobility, worsening the Karnofsky Performance Status (KPS). Surgery for spinal metastases has the potential to improve KPS and extend prognosis, but it is crucial to recognize the elevated risk of perioperative complications. Therefore, the development of a new scoring system to accurately predict perioperative complications in spinal metastatic surgery is essential.

**Methods:**

We conducted a retrospective observational study with 86 patients who underwent surgical intervention for spinal metastases. Patients were divided into two groups based on the presence or absence of perioperative complications within 14 days after surgery. Various factors related to perioperative complications were assessed through univariate and multivariate analyses. We established a clinical prognostic scoring system called the Perioperative Complications following Metastatic Spinal Surgery (PERCOM) score and evaluated its precision using receiver operating characteristic (ROC) analysis.

**Results:**

Five variables (age, KPS, primary prostate cancer, Albumin, and Hemoglobin) identified in the univariate analysis were assigned binary values of 0 or 1. The PERCOM score was then calculated for each patient by summing the individual points, ranging from 0 to 5. The optimal threshold determined by ROC curve analysis for the PERCOM score was 2 points, with a sensitivity of 86 % and a specificity of 56 %.

**Conclusions:**

The composite PERCOM score effectively predicted perioperative complications in spinal metastasis surgery. To further validate its precision, a prospective multicenter study is needed.

## 1 Introduction

Recent advancements in cancer therapy have significantly extended the life expectancy of patients with bone metastases, including spinal metastasis [1]. Spinal metastases can lead to spinal cord compression and intractable pain, ultimately resulting in the loss of ambulatory function [2]. Meanwhile, the concept of Karnofsky Performance Status (KPS), which assesses ambulatory capability and patient independence, holds great importance in cancer treatment. Notably, patients with a KPS score of 40 or lower, indicating limited ambulatory function and independence, often do not qualify for participation in clinical trials or aggressive treatment [3]. Surgery for spinal metastases plays a pivotal role in preserving neurological function and alleviating intractable pain [4]. Consequently, one of the key objectives of spinal metastasis surgery is to enhance KPS and prolong prognosis through continuous treatment.

Performing surgery for spinal metastases presents inherent challenges and necessitates the involvement of a skilled multidisciplinary team [5]. Additionally, this type of surgical intervention carries a substantial perioperative morbidity risk, ranging from 10 % to 76 % [6]. Such complications can lead to treatment delays, underscoring the importance for spine surgeons to thoroughly weigh the benefits and risks of surgical intervention, particularly in cases with poor preoperative functional status or limited expected survival. However, current scoring systems for perioperative complications following spinal metastasis surgery remain limited [[7], [8], [9]]. Therefore, the aim of this study was to develop a novel scoring system to accurately predict the risk of perioperative complications in spinal metastasis surgery.

## 2 Methods

We conducted a retrospective cohort study comprising 86 patients diagnosed with spinal metastases who underwent surgical intervention at our department from 2012 to 2021. All patients underwent palliative posterior decompression coupled with posterior fusion surgery using either percutaneous or conventional approaches. The cases employing percutaneous approaches were specifically classified as minimally invasive spinal stabilization (MISt). Patients were stratified into two cohorts based on the occurrence or non-occurrence of perioperative complications in order to identify the risk factors associated with such complications. The study received approval from the institutional review board at Kyushu University Hospital (26–112).

### 2.1 Definition of perioperative complications

Perioperative complications were examined utilizing the Clavien-Dindo classification. Cases experiencing Clavien-Dindo grade two or higher complications within 14 days post-surgery were regarded as having complications. Within this classification, grade 2 or higher complications were characterized as potentially life-threatening, necessitating therapeutic intervention or a hospitalization duration exceeding twice the median length of stay for the corresponding illness [10].

### 2.2 Outcome measures

#### 2.2.1 Patients-related factors

Patient-related factors encompassed variables such as age, gender, presence of diabetes mellitus (DM), KPS, body mass index (BMI), Tokuhashi score [11], albumin (Alb) level, hemoglobin (Hb) level, and modified Glasgow prognostic score (mGPS) [12] measured immediately prior to surgery.

#### 2.2.2 Disease-related factors

Disease-related factors included patients with primary prostate cancer, the number of affected vertebrae, preoperative ambulatory dysfunction, as well as bladder and rectal disorders.

#### 2.2.3 Preoperative treatment

Patients who received preoperative treatment, including radiation therapy, pharmacotherapy, molecularly targeted drugs, angiogenesis inhibitors, hormone therapy, or immune checkpoint inhibitors, were included in the analysis, regardless of the specific time frame.

#### 2.2.4 Surgery-related factors

Surgery-related factors comprised patients who underwent MISt, estimated blood loss, surgeries lasting over 5 h (defined based on a prior study [13]), and preoperative embolization.

#### 2.2.5 Statistical analysis

Outcome measures were subjected to statistical analysis, encompassing means and standard deviations for continuous variables, as well as frequencies and percentages for categorical variables. Following a descriptive analysis of demographics, the association between preoperative variables and the occurrence of complications was examined through univariate analyses. Subsequently, potential risk factors (p < 0.1) were selected and analyzed using multivariate logistic regression analyses. Statistical significance was determined at P < 0.05. To assess the model's accuracy in predicting perioperative complications, optimal cut-off points for the score were determined by analyzing receiver operating characteristic (ROC) curves and selecting the point nearest to the upper left corner of the ROC graph. Statistical analysis was performed using JMP version 14 software, SAS Institute, Cary, USA.

## 3 Results

### 3.1 Patients’ demographics

A total of 86 consecutive cases were included in this study, with a mean age of 64.3 years (range: 37–86 years). Among the participants, there were 51 males and 35 females. Twelve patients had DM. All patients underwent palliative decompressive surgery.

### 3.2 Perioperative complications

Among the 87 patients, 15 (17 %) experienced perioperative complications. The breakdown of complications, accounting for duplicates, included wound dehiscence in 7 cases (8.0 %), wound infection in 6 cases (6.9 %), deep vein thrombosis in 4 cases (4.6 %), hematoma in 2 cases (2.3 %), pulmonary embolism in 1 case (1.1 %), disseminated intravascular coagulation in 1 case (1.1 %), perforation of the digestive tract in 1 case (1.1 %), and pneumonia in 1 case (1.1 %). Notably, the most prevalent complications were observed in patients with prostate cancer (4 cases), while the remaining primary sites accounted for one case each.

### 3.3 Risk factors of complications in spine metastasis surgery

#### 3.3.1 Univariate analysis

Significant differences (p < 0.05) were noted in age and KPS between the two groups. Additionally, patients with perioperative complications tended to have lower levels of Alb and Hb (p < 0.1) (Table 1). Among the tumor-related factors, primary prostate cancer exhibited significant differences, while the number of affected vertebrae, preoperative ambulatory disability, and bladder bowel dysfunction did not significantly differ between the two groups (Table 2). Preoperative treatments, such as radiation therapy, drug therapy, molecular-targeted drugs, angiogenesis inhibitors, hormone therapy, and immune checkpoint inhibitors, did not show significant differences with or without perioperative complications (Table 3). Furthermore, there were no significant differences between the two groups in any of the surgery-related factors, including MISt, blood loss, prolonged surgery longer than 5 h, and preoperative embolization (Table 4).

**Table 1:** Patients-related factors.

General condition	Complication (n = 15)	No Complication (n = 71)	p-value
Age (y/o)	68.7 (37–87)	61.6 (22–88)	0.0453
Sex (male/female)	11/4	40/31	n.s.
DM	4	8	n.s.
KPS	36 (20–70)	44 (20–90)	0.0348
BMI	20.4 (16.2–31.2)	21.7 (15.3–27.6)	n.s.
Tokuhashi score	6.7 (3–10)	6.1 (0–13)	n.s.
Alb	3.5 (2.8–4.6)	3.75 (2.1–4.8)	0.087
Hb	11.6 (8.7–14.0)	12.6 (8.4–16.8)	0.064
modified Glasgow Prognostic Score	1.06	0.76	n.s

DM: diabetes mellitus.

KPS: Karnofsky performance status.

BMI: body mass index.

Alb: serum albumin.

Hb: hemoglobin.

n.s.: not significant.

**Table 2:** Tumor-related factors.

Tumor-related factors	Complication (n = 15)	No Complication (n = 71)	p-value
primary prostate cancer	4 out of 7	3 out of 7	0.015
number of affected vertebrae	3.45 (1–8)	3.13 (1–9)	n.s.
preoperative inability to walk	6	18	n.s.
bladder and rectal disorders	9	22	n.s.

n.s.: not significant.

**Table 3:** Preoperative treatment.

Preoperative treatment	Complication (n = 15)	No Complication (n = 71)	p-value
radiation therapy	5	26	n.s.
drug therapy	11	45	n.s.
molecularly targeted drugs	6	17	n.s.
angiogenesis inhibitors	3	6	n.s.
hormone therapy	3	6	n.s.
immune checkpoint inhibitors	1	4	n.s.

n.s.: not significant.

**Table 4:** Surgery-related factors.

Surgery-related factors	Complication (n = 15)	No Complication (n = 71)	p-value
MISt	2	16	n.s
Estimate blood loss (mL)	663 (55–2400)	496 (little-5549)	n.s.
prolonged surgery longer than 5 h	2	4	n.s.
preoperative embolization	2	3	n.s.

MISt: minimally invasive spine stabilization.

n.s.: not significant.

#### 3.3.2 Multivariate analysis

Five factors that demonstrated a strong association (p < 0.1) with perioperative complications in the univariate analysis were included in the multivariate analysis. However, no individual factor showed a significant association with perioperative complications in spine metastasis surgery (Table 5).

**Table 5:** Multivariate analysis.

	Odds ratio	95 % Confidence interval	p-value
age	1.04	0.99–1.1	0.1
KPS	0.96	0.91–1.0	0.12
primary prostate cancer	3.54	0.57–23.8	0.17
Alb	0.83	0.19–3.67	0.8
Hb	0.78	0.5–1.18	0.24

KPS: Karnofsky performance status.

Alb: serum albumin.

Hb: hemoglobin.

### 3.4 Development of clinical prediction score for perioperative complications following metastatic spinal surgery (PERCOM)

**Fig. 1:**
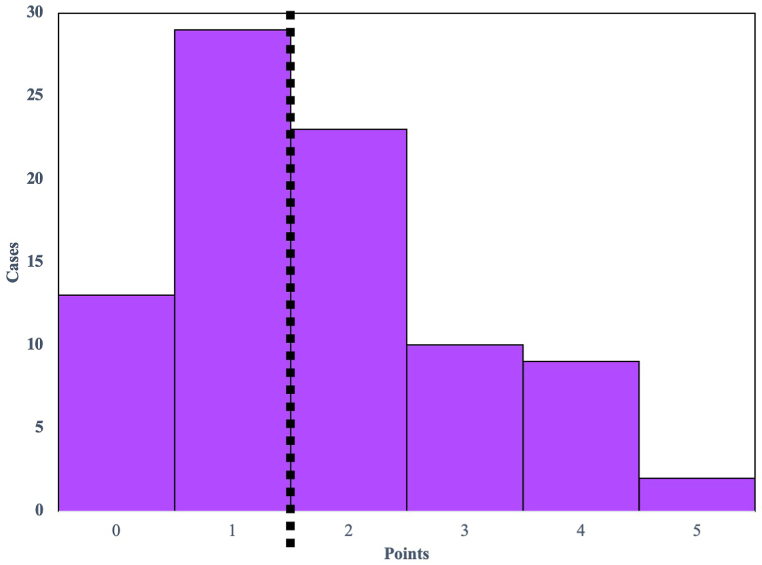
Histogram depicting the distribution of PERCOM scores. The optimal threshold for the PERCOM score, determined through ROC curve analysis, was identified as 2 points.

**Fig. 2:**
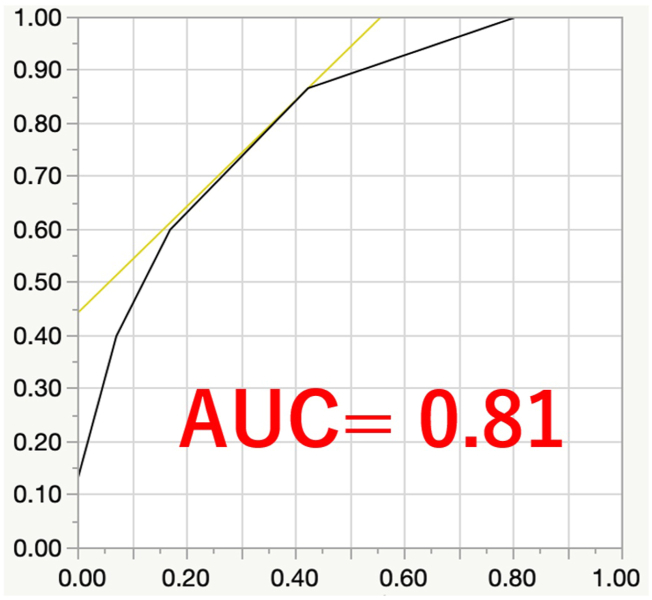
ROC curve analysis was performed to evaluate the predictive capacity of the PERCOM score. The AUC was determined to be 0.81, indicating a high level of accuracy. Additionally, based on the ROC curve analysis, the optimal threshold for the PERCOM score was identified as 2 points.

To predict perioperative complications following metastatic spinal surgery, we developed a new and simplified scoring system based on the significant risk factors (p < 0.1) identified in the univariate analysis. The five parameters were assigned a value of 0 if absent or 1 if present, and the clinical prediction score for perioperative complications following metastatic spinal surgery (PERCOM score) was calculated for each case by summing the total number of points, ranging from 0 to 5 (Table 6). The distribution of the PERCOM score is illustrated in Fig. 1. The area under the ROC curve (AUC) for the PERCOM score was 0.81, indicating a highly accurate prediction of the occurrence of complications (Fig. 2). Additionally, the optimal cut-off value for the PERCOM score was determined to be 2 points. Based on this value, the sensitivity and specificity for predicting perioperative complications were 86 % and 56 %, respectively.

**Table 6:** PERCOM score.

	cut off	score
age	over 69 y/o	1
KPS	under 40	1
primary prostate cancer	yes	1
Alb	under 3.8	1
Hb	under 12.1	1

KPS: Karnofsky performance status.

Alb: serum albumin.

Hb: hemoglobin.

## 4 Discussion

Multiple studies have been conducted to develop scoring systems for predicting perioperative complications in spinal metastasis surgery. Schoenfield et al. proposed the New England Spinal Metastasis Score (NESMS), which aids in predicting 30-day morbidity following surgery for spinal metastases [7]. The study included a total of 776 patients. The NESMS assigns points based on three factors: a patient's modified Bauer score [14] (≤2 vs. ≥3), functional status (ambulatory or non-ambulatory), and preoperative serum albumin level (<3.5 g/dL vs. ≥3.5 g/dL). The maximum score achievable on the NESMS is 3. The researchers reported that the AUC for major complications was 0.69.

Furthermore, Ramos et al. have devised the Metastatic Spinal Frailty Index (MSTFI) based on a comprehensive analysis of a nationwide database in the USA [8]. The MSTFI incorporates nine factors: anemia, chronic lung disease, coagulopathy, electrolyte abnormalities, pulmonary circulation disorders, renal failure, malnutrition, emergent/urgent admission, and anterior/combined surgical approach. The MSTFI has a maximum score of 10, and patients are categorized based on their score: 0 points as “not frail,” 1 point as “mildly frail,” 2 points as “moderately frail,” and 3 or more points as “severely frail.” Comparatively, patients with mild, moderate, and severe frailty demonstrated significantly higher odds of experiencing perioperative complications than those without frailty (all P < 0.001). The researchers also calculated an AUC of 0.67 for predicting perioperative complications.

Meanwhile, in 2020, Ramos et al. introduced the Spine Oncology Morbidity Assessment (SOMA) score, which represents a significant advancement in the field [9]. The SOMA score incorporates five variables: age exceeding 70 years, hypoalbuminemia, compromised functional status, Frankel classification, and the number of affected vertebrae (greater than 2 continuous vertebral bodies). Ranging from 0 to 5 points, this score underwent validation in a cohort of 105 patients, demonstrating an impressive AUC of 0.75 for predicting postoperative complications.

Common elements in both our scoring system and the aforementioned scoring systems include low albumin levels and impaired ambulation. Notably, all of these elements are closely linked to frailty [15]. Conversely, preoperative treatment and surgery-related factors were not identified as significant risk factors. This observation suggests that the occurrence of perioperative complications in spinal metastasis surgery is heavily influenced by the patient's underlying condition, particularly their level of frailty. Therefore, it is crucial to consider, prior to surgery, that the risk of perioperative complications following surgery for metastatic spinal tumors is substantially elevated in patients whose general condition has declined due to disease progression or chemotherapy-related side effects.

The present study differs from previous investigations by identifying primary tumor location, specifically prostate cancer, as a noteworthy risk factor. Typically, spinal metastases originating from prostate cancer manifest as osteogenic metastases, which seldom necessitate surgical intervention [16]. Consequently, it is anticipated that the disease has often progressed significantly by the time surgery becomes necessary. In a meta-analysis focusing on spinal metastases in prostate cancer, Clarke et al. reported a postoperative complication rate of 32 %, suggesting that the complication rate may be higher compared to other primary tumor sites [17]. Notably, in this study, patients with prostate cancer exhibited significantly lower KPS scores compared to patients with cancer originating from other locations prior to surgery. Thus, patients with spinal metastases from prostate cancer who undergo surgery are more likely to experience frailty, consequently leading to a higher complication rate.

The limitations of this study encompass its retrospective nature and the inclusion of a small patient cohort, potentially introducing significant selection bias. Moreover, the lack of external validation for the PERCOM score also represents a limitation. To address these shortcomings, we plan to establish a multicenter database that would provide an opportunity to overcome these limitations. Subsequently, a prospective multicenter study will be imperative to validate the accuracy of our findings and generate robust evidence for future clinical practice.

In conclusion, the proposed PERCOM score demonstrates notable sensitivity as a predictive tool for perioperative complications following spinal metastatic surgery. Incorporating the PERCOM score into the decision-making process for complex cases of spinal metastasis may significantly enhance clinical judgment. In cases where the score falls within the high range (3–5), conventional perioperative treatment strategies may prove inadequate. As wound complications are particularly prevalent in surgery for metastatic spinal tumors, considering preventive measures such as frequent wound cleansing, subcutaneous irrigation prior to wound closure, and unconventional antibiotic administration methods may be advisable.

## Ethics statement

The study was reviewed and approved by the institutional review board at the Kyushu University Hospital (26–112). Since this study included patients who had attended our clinic in the past and it was difficult to obtain informed consent again, the opt-out method was applied to obtain patient consent.

## Data availability statement

The datasets for this study are available on reasonable request to the corresponding author.

## Funding

The author(s) disclosed receipt of the following financial support for the research, authorship, and/or publication of this article: This work was supported by a Grant-in-Aid for Scientific Research from the 10.13039/100016626Japanese Orthopaedic Association (JOA- Subsidized Science Project Research 2022-1).

## CRediT authorship contribution statement

**Ryouhei Takeuchi:** Data curation, Formal analysis, Investigation, Writing – original draft. **Kiyoshi Tarukado:** Investigation, Methodology, Writing – review & editing. **Yoshihiro Matsumoto:** Data curation, Formal analysis, Funding acquisition, Methodology, Writing – review & editing. **Kei-ichiro Iida:** Supervision. **Kazu Kobayakawa:** Supervision. **Hirokazu Saiwai:** Supervision. **Kenichi Kawaguchi:** Supervision. **Yasuharu Nakashima:** Supervision.

## Declaration of competing interest

The authors declare that they have no known competing financial interests or personal relationships that could have appeared to influence the work reported in this paper.
