# Supplementary material for: Development of a clinical prediction score for perioperative complications following metastatic spinal surgery (PERCOM) score
Source: Heliyon. 2024 Jan 26;10(3):e25180. doi: 10.1016/j.heliyon.2024.e25180 (PMC10850538; doi:10.1016/j.heliyon.2024.e25180)
Supplement: Multimedia component 1 [file mmc1.pdf]

平成２６年７月３１日

申請者
九州大学大学院医学研究院　整形外科学分野
教授　　岩本　幸英　殿

九州大学病院長
石　橋　達　朗　公印省略

九州大学医系地区部局臨床研究倫理審査委員会における審査結果について（通知）

貴殿から申請がありました下記の研究について、九州大学医系地区部局臨床研究倫理審査委員会における審査結果に基づき検討した結果、その実施を許可します。

研究の実施にあたり、研究計画書を遵守の上、毎年一回、及び研究の終了時若しくは中止時に、研究実施報告書を提出願います。

また、研究実施中に、個人情報保護に係る不利益が生じたとき、または健康被害等の有害事象が生じた場合には、直ちに報告してください。

記

判　　定	許可
許可番号	２６－１１２
課 題 名	脊柱変形、腫瘍性疾患の画像所見に関する研究
許可期間	平成２６年７月３１日　～　平成３１年４月３０日
備　　考	
