# Supplementary material for: Development of a clinical prediction score for perioperative complications following metastatic spinal surgery (PERCOM) score
Source: Heliyon. 2024 Jan 26;10(3):e25180. doi: 10.1016/j.heliyon.2024.e25180 (PMC10850538; doi:10.1016/j.heliyon.2024.e25180)
Supplement: Multimedia component 2 [file mmc2.pdf]

Ver. 1.1

平成　　年　　月　　日

□　大学院医学研究院長
□　大学院歯学研究院長
□　大学院薬学研究院長　　　殿
□　生体防御医学研究所長
☑　九 州 大 学 病 院 長

所　属　九州大学整形外科
申請者 職　名　教授　　　　印
氏　名　岩本　幸英

倫理審査申請書

下記について、審査を申請します。

1．課題名　脊柱変形、腫瘍性疾患の画像所見に関する研究		
2．研究責任者所属・職・氏名　整形外科　教授　岩本幸英	認定番号	０００００２８
2．研究分担者所属・職・氏名<br>九州大学病院　整形外科　講師　播广谷勝三<br>九州大学病院　整形外科　助教　松本嘉寛<br>九州大学病院　リハビリテーション科　助教　林田光正	認定番号	０００６８４<br>００１０２２<br>００２５９０
九州大学病院　病理診断科・病理部　部長　小田義直	認定番号	０００２９２
4．多施設共同研究の場合（他施設の研究者の所属・職・氏名）		
5．研究デザイン（研究計画に基づき、次のいずれかを○で囲んでください。）<br>・観察研究（○）　　・介入研究		
6．研究計画の概要<br>脊柱変形、腫瘍性疾患は、脊柱支持機構および脊髄麻痺を呈する疾患である。最近、手術療法の進歩により、治療成績は向上してきたが、依然、疼痛、麻痺により生活が制限される症例も少なくない。また、合併症をきたす症例も多く、その対策や治療について検討を行うことはいずれも治療向上に重要である。更に、脊椎疾患には様々なものがあり、鑑別に苦慮することも多い。従って、その診断や治療方法について画像所見を中心に解析し、臨床成績との検討を行う予定である。		
7．研究の実施場所　九州大学臨床研究A棟5階 整形外科学教室<br>九州大学病院整形外科病棟および整形外科外来		
8．研究期間（研究期間は５年以内とすること。）<br>承認日　～　平成 31年　4月　30日		
9．研究経費：種類（文部科学（○）・厚生労働省科学研究費、財団研究費、寄附金、その他（　　　））<br>＊重複可能　　　金額　416万円（期間:　　　　）		
10．利益相反：　有　無（○）		
11．高度先進医療の申請：　有　無（○）		
12．インフォームド・コンセント取得の有無<br>無（○）(情報公開の方法：ホームページにて　　　)<br>有（文書　口頭［記録の方法：　　　］　）		
13．個人情報管理責任者所属・職・氏名　医学研究院臨床医学部門整形外科学分野<br>教授　岩本幸英		

14．研究等の情報の公開（次の一つを○で囲むこと。）　全面公開可　　部分公開可　　公開不可<br>＊公開不可の場合は、委員会で理由を述べること。
部分公開の場合、公開できる部分を○で囲むこと。<br>①研究課題名　　②申請者・説明者・同席者の所属・職名・氏名　　③審査結果
